# Sensitive ELISA-based detection method for the mitophagy marker p-S65-Ub in human cells, autopsy brain, and blood samples

**DOI:** 10.1080/15548627.2020.1834712

**Published:** 2020-10-28

**Authors:** Jens O. Watzlawik, Xu Hou, Dominika Fricova, Chloe Ramnarine, Sandeep K. Barodia, Tania F. Gendron, Michael G. Heckman, Michael DeTure, Joanna Siuda, Zbigniew K. Wszolek, Clemens R. Scherzer, Owen A. Ross, Guojun Bu, Dennis W. Dickson, Matthew S. Goldberg, Fabienne C. Fiesel, Wolfdieter Springer

**Affiliations:** a Department of Neuroscience, Mayo Clinic, Jacksonville, FL, USA; b Center for Neurodegeneration and Experimental Therapeutics, The University of Alabama at Birmingham, Birmingham, AL, USA; c Neuroscience PhD Program, Mayo Clinic Graduate School of Biomedical Sciences, Jacksonville, FL, USA; d Division of Biomedical Statistics and Informatics, Mayo Clinic, Jacksonville, FL, USA; e Department of Neurology, Medical University of Silesia, Katowice, Poland; f Department of Neurology, Mayo Clinic, Jacksonville, FL, USA; g Center for Advanced Parkinson Research, Harvard Medical School, Brigham and Women’s Hospital, Boston, MA, USA; h Department of Neurology, Department of Neurobiology, The University of Alabama at Birmingham, Birmingham, AL, USA

**Keywords:** Alzheimer disease, autophagy, mitophagy, Parkin, Parkinson disease, PINK1, PRKN, ubiquitin

## Abstract

Mitochondrial dysfunction is an early, imminent event in neurodegenerative disorders including Parkinson disease (PD) and Alzheimer disease (AD). The enzymatic pair PINK1 and PRKN/Parkin recognize and transiently label damaged mitochondria with ubiquitin (Ub) phosphorylated at Ser65 (p-S65-Ub) as a signal for degradation via the autophagy-lysosome system (mitophagy). Despite its discovery in cell culture several years ago, robust and quantitative detection of altered mitophagy *in vivo* has remained challenging. Here we developed a sandwich ELISA targeting p-S65-Ub with the goal to assess mitophagy levels in mouse brain and in human clinical and pathological samples. We characterized five total Ub and four p-S65-Ub antibodies by several techniques and found significant differences in their ability to recognize phosphorylated Ub. The most sensitive antibody pair detected recombinant p-S65-Ub chains in the femtomolar to low picomolar range depending on the poly-Ub chain linkage. Importantly, this ELISA was able to assess very low baseline mitophagy levels in unstressed human cells and in brains from wild-type and *prkn* knockout mice as well as elevated p-S65-Ub levels in autopsied frontal cortex from AD patients vs. control cases. Moreover, the assay allowed detection of p-S65-Ub in blood plasma and was able to discriminate between *PINK1* mutation carriers and controls. In summary, we developed a robust and sensitive tool to measure mitophagy levels in cells, tissue, and body fluids. Our data strongly support the idea that the stress-activated PINK1-PRKN mitophagy pathway is constitutively active in mice and humans under unstimulated, physiological and elevated in diseased, pathological conditions.

**Abbreviations**: Ab: antibody; AD: Alzheimer disease; AP: alkaline phosphatase; CV: coefficient of variation; ECL: electrochemiluminescence; KO: knockout; LoB: Limit of Blank; LoD: Limit of Detection; LoQ: Limit of Quantification; MSD: meso scale discovery; PD: Parkinson disease; p-S65-PRKN: phosphorylated PRKN at serine 65; p-S65-Ub: phosphorylated ubiquitin at serine 65; Std.Dev.: standard deviation; Ub: ubiquitin; WT: wild type

## Introduction

In the management of Parkinson disease (PD) and other neurodegenerative disorders, robust diagnostic and prognostic biomarkers are urgently needed to identify early disease stages before the onset of irreversible neuropathological changes. This includes an estimated loss of 30–50% dopaminergic neurons in the substantia nigra and a loss of 70–80% dopaminergic terminals in the striatum, which are responsible for early symptoms in PD [1–4] Corresponding morphological changes in Alzheimer disease (AD) are brain atrophy that result in mild to moderate cognitive impairment [5]. The identifications of early disease stages in both PD and AD, during which patients may benefit the most from prospective disease-modifying drugs, are most likely missed by the existing diagnostic based on motor or cognitive symptoms [6,7]. While reliable biomarkers of early neurodegenerative stages are currently lacking, a recent liquid chromatography-mass spectrometry study identified multiple abnormal changes in metabolite levels in the plasma of PD patients. Of note, all changes were related to lipid metabolism and mitochondrial function [8].

Mitochondrial dysfunctions as well as impairments of the autophagic-lysosomal degradation systems are indeed amongst the earliest cellular alterations and are detectable years before the deposition of the respective aggregation-prone proteins [9–11]. Loss of function mutations in the *PINK1* (PTEN induced kinase 1) and *PRKN* genes are the most common causes of early-onset PD [12,13]. Functional studies demonstrate that both encoded enzymes together orchestrate a protective mitochondrial quality control [14–22] and thereby also regulate innate and adaptive immunity [23,24]. While the kinase PINK1 is continuously imported into healthy mitochondria and degraded, PINK1 accumulates on the surface of damaged organelles where it phosphorylates a conserved serine 65 residue of both the small modifier ubiquitin (Ub) [25–27] and the E3 Ub ligase PRKN/Parkin [20,28–30]. Both phosphorylations lead to the recruitment and full activation of PRKN. Fully activated PRKN together with PINK1 then decorate mitochondrial outer membrane proteins with phosphorylated Ub (p-S65-Ub) through a feed-forward mechanism [31–33]. Though only transient under normal conditions, this mitophagy tag significantly increases with stress, age, and disease and as such is a pathophysiological relevant marker [34–36].

Here we developed a meso-scale discovery (MSD)-based sandwich ELISA to measure p-S65-Ub with the goal to assess both baseline and diseased mitophagy levels from clinical and pathological human specimens. It is of note that while PINK1-PRKN-mediated mitophagy is well established under cell culture conditions using high concentrations of non-physiological mitochondrial depolarizers, the extent of activation and mitochondrial turnover *in vivo* under non-diseased, unstimulated conditions as well as under pathological conditions and particularly in mice are still controversial [37]. Importantly, changes in the mitophagy marker p-S65-Ub are dynamic in nature and levels may be elevated due to either increased mitochondrial stress and/or impaired mitochondrial turnover through the autophagic-lysosomal system. Abnormal p-S65-Ub levels could indicate or complement existing criteria for early diagnosis of PD or AD and may also serve as a prognostic marker in different neuropathological diseases. Besides its relevance as a potential biomarker, monitoring of p-S65-Ub levels might also be utilized to measure pharmacodynamics in response to future therapeutics aiming to restore mitophagy flux.

## Results

### Western blot assessment of total Ub, p-S65-Ub, and p-S65-PRKN

**Figure 1.**
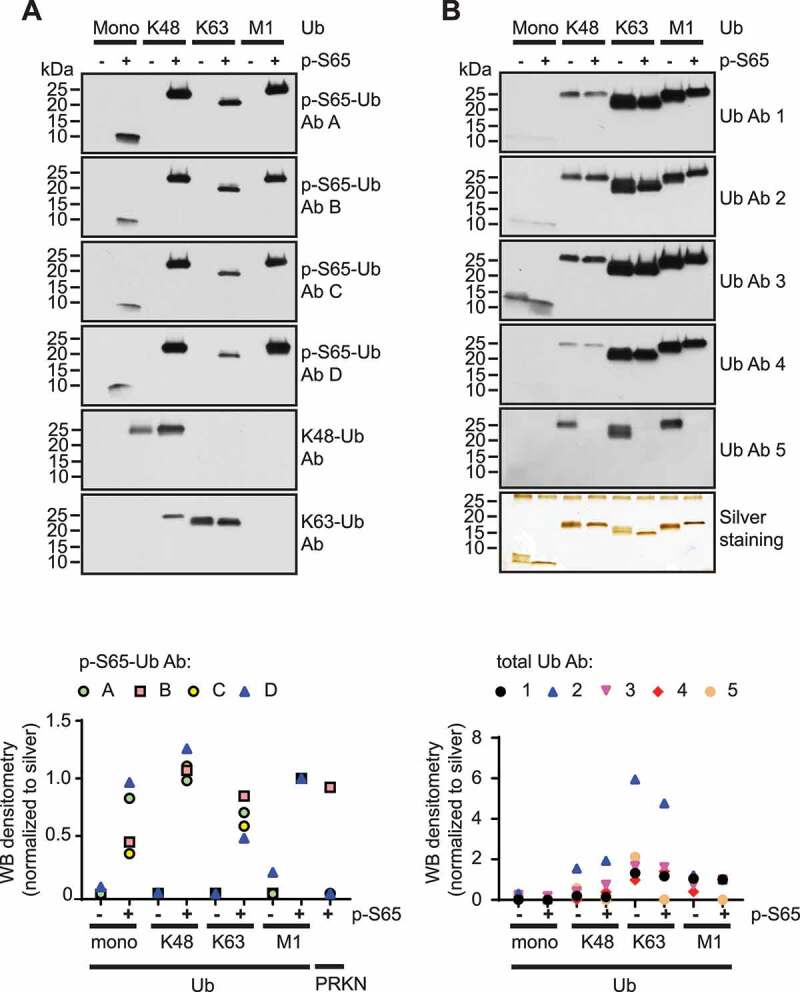
Western blot assessment of phosphorylated Ub species. Equal masses of recombinant Ub and p-S65-Ub monomers and tetramers with different chain linkages were tested. Shown are representative western blots (top panels) and quantifications relative to silver stained gels (bottom panels). (A) Membranes were probed with four different p-S65-Ub Abs (A-D) and linkage-specific poly-Ub Abs targeting K48- or K63 chains. (B) Membranes were probed with five different total Ub Abs (1–5) (see Table 1). Western blot intensity levels were normalized to intensity levels from corresponding silver staining

To evaluate specificities of different antibodies (Abs), we first tested recombinant Ub and p-S65-Ub monomers and tetramers (Ub_4_) with different chain linkages in western blots. We chose K48 and K63 linked poly-Ub chains that are amongst the most abundant signals during mitophagy as well as linear, M1-linked chains as additional controls [38]. Fourfold molar excess of Ub over Ub_4_ was used to compensate for additional epitopes in the tetramers over the monomers. The four rabbit p-S65-Ub Abs #A-D (Table 1) tested showed high specificity for phosphorylated versus non-phosphorylated Ub species (Figure 1A). Quantification of detected signals on western blots after normalization to the corresponding silver stained proteins revealed the following affinities toward p-S65-Ub species: K48-linked p-S65-Ub_4_ > M1-linked p-S65-Ub_4_ > K63-linked p-S65-Ub_4_ > p-S65-Ub monomers. All four p-S65-Ub Abs clustered tightly for each linkage-specific p-S65-Ub_4_ with more diversity amongst their ability to target p-S65-Ub monomers. Of the four Abs tested, only p-S65-Ub Ab B cross reacted with recombinant p-S65-PRKN protein with intensity levels similar to those obtained for M1-linked p-S65-Ub_4_ (Fig. S1).

**Table 1.** Primary and secondary Abs used in the study

Text identifier	Clone	Dilution WB (Final conc.)	MSD (Final conc. in μ/ml)	ICC/IHC (Final conc. in μg/ml)	Host Species	Company	Catalog No.
p-S65-Ub Ab A	Polyclonal	1:1,000 (0.24 μg/mL)	1	1.9 /4.7	Rabbit IgG	CST	37642
p-S65-Ub Ab B	Polyclonal	1:1,000 (0.51 μg/mL)	1	2.0 /0.77	Rabbit IgG	Springer-lab	in house Ab
p-S65-Ub Ab C	Polyclonal	1:1,000 (0.5 μg/mL)	1	2.0 /10	Rabbit IgG	Millipore	ABS1513
p-S65-Ub Ab D	E2J6T	1:1,000 (0.1 μg/mL)	1	0.08 /0.4	Rabbit IgG	CST	62802
Ub Ab 1	1D7B2	1:1,000 (0.65 μg/mL)	1	– -	Mouse IgG1	Proteintech Group	60310-1-01 g
Ub Ab 2	P4D1	1:1,000 (0.2 μg/mL)	0.2, 1, 5 μg/ml	– -	Mouse IgG1	Santa Cruz	sc-8017
Ub Ab 3	UBI-1	1:1,000 (1 μg/mL)	1 μg/ml	– -	Mouse IgG1	Millipore	MAB1510
Ub Ab 4	VU-1	1:1,000 (0.5 μg/mL)	1 μg/ml	– -	Mouse IgG1	Life Sensors	VU-101
Ub Ab 5	FK2	1:1,000 (1 μg/mL)	1 μg/ml	– -	Mouse IgG1	Millipore	04–263
K48-Ub Ab	D9D5	1:1,000	– -	– -	Rabbit IgG	CST	8081
K63-Ub Ab	D7A11	1:1,000	– -	– -	Rabbit IgG	CST	5621
p-S65-PRKN	polyclonal	1:5,000	– -	– -	Rabbit IgG	Muqit-lab	– –
PRKN Ab	Prk8	1:2,000	– -	– -	Mouse IgG1	CST	4211
PINK1 Ab	D8G3	1:1,000	– -	– -	Rabbit IgG	CST	6946
GAPDH Ab	– -	1:400,000	– -	– -	Mouse IgG1	Meridian	H86504M
Anti-mouse IgG, HRP-linked	Secondary Ab	1:10,000	– -	– -	Donkey IgG	Jackson IR	715–035-150
Anti-rabbit IgG, HRP-linked	Secondary Ab	1:10,000	– -	– -	Donkey IgG	Jackson IR	711–035-152

WB: western blot; MSD: Meso Scale Discovery; ICC: immunocytochemistry; IHC: immunohistochemistry; CST: Cell Signaling Technology; Jackson IR: Jackson ImmunoResearch Laboratories, Inc.; Santa Cruz: Santa Cruz Biotechnology

In contrast to p-S65-Ub Abs, the five mouse total Ub Abs #1-5 (Table 1) generally detected non-phosphorylated Ub similarly to phosphorylated Ub, with the exception of Ub Ab 5, which exclusively recognized non-phosphorylated Ub_4_ (Figure 1B). There was a preference of all five total Ub Abs to recognize K63-linked chains over M1- and K48-linked Ub_4_ or Ub monomers. Comparing the different total Ub Abs with each other revealed the highest signal intensity per area for Ub Ab 2 > Ub Ab 1 > Ub Ab 3 > Ub Ab 4 > Ub Ab 5. In summary, western blot assessment of Abs targeting p-S65-Ub and total Ub species revealed binding preferences for specific Ub chain linkages as well as molecule size (Ub monomers vs. tetramers).

### Validation of the p-S65-Ub Abs specificity in cells and tissue

**Figure 2.**
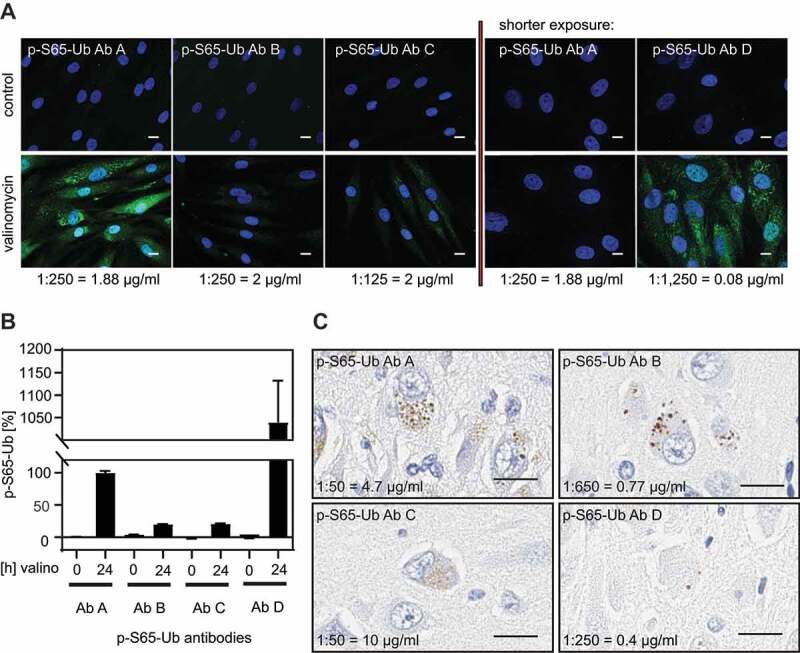
Validation of p-S65-Ub antibodies in fixed human cells and autopsy brain tissue. (A) All four p-S65-Ub Abs were evaluated by immunocytochemistry of human control skin fibroblasts with or without valinomycin (1 μM) treatment for 24 h. p-S65-Ub Abs A-C were tested first in similar concentrations showing positive (green) staining only for p-S65-Ub Ab A in valinomycin-treated fibroblasts (left). Due to its higher binding affinity, p-S65-Ub Ab D was compared only to p-S65-Ub Ab A at ~25 fold higher Ab dilution relative to the initial set using p-S65-Ub Abs A-C (right). Scale bar: 10 μm. (B) Fluorescence intensities were then quantified by high content imaging and compared relative to levels obtained with p-S65-Ub Ab A. (C) All four p-S65-Ub Abs were further analyzed by immunohistochemistry using 5-micron sections of paraffin-embedded hippocampal AD postmortem brain tissue. Scale bar: 20 μm

To further confirm the specificity of the p-S65-Ub signal from cells and tissue, we next validated the p-S65-Ub Abs by additional, visual methods. First, human control skin fibroblasts were treated with or without valinomycin, labeled with p-S65-Ub Abs A-D, and processed for high content imaging (Figure 2A). For each of the Abs, we selected concentrations to sufficiently label intracellular pools of phosphorylated Ub upon mitochondrial depolarization. Only very weak background signal was detected in untreated cells confirming the specificity. As for p-S65-Ub Ab D, a 25-fold lower final Ab concentration compared to p-S65-Ub Abs A-C (0.08 μg/ml compared to ~2 μg/ml) already achieved strong staining by immunocytochemistry. Quantification of cytoplasmic fluorescent signal with high content imaging confirmed that p-S65-Ub Ab D showed 10 times higher signal compared to p-S65-Ub Ab A with the overall intensity following the order: p-S65-Ub Ab D > p-S65-Ub Ab A > p-S65-Ub Ab B and p-S65-Ub Ab C (Figure 2B).

In analogy to staining of fibroblasts in culture by immunocytochemistry, we assessed p-S65-Ub labeling in human autopsy brain (Figure 2C). Hippocampal sections were stained with p-S65-Ub Abs A-D followed by diaminobenzidine and nuclear hematoxylin counterstaining. This revealed small round, cytoplasmic structures similar to the previously reported, granular p-S65-Ub morphologies [34,36,39]. Comparable staining intensities were achieved when different dilutions were used for the four p-S65-Ub Abs. Taken together, while the four p-S65-Ub antibodies appear to have different affinities in immunostaining they detect the same characteristic morphologies upon mitochondrial stress in cells and in human disease autopsy brain.

### Sandwich ELISA p-S65-Ub assessment using different Ab combinations

Given that the binding preferences for total Ub and p-S65-Ub Abs were rather opposite, predicting the optimal Ab combination to be used in sandwich-type ELISAs was challenging. We therefore tested all four rabbit p-S65-Ub Abs #A-D as capturing agents each combined with five different mouse total Ub Abs #1-5 (Table 1) as detecting tools in sandwich ELISAs for the assessment of p-S65-Ub and ran them on the Meso Scale Discovery (MSD) platform that uses electrochemiluminescence (ECL) as a readout. Analogous to the western blots, we first tested recombinant p-S65-Ub monomers and tetramers with different chain linkages as analyte and used their non-phosphorylated counterparts as negative controls. Identical concentrations rather than equimolar quantities were chosen to compensate for fourfold higher epitope numbers in Ub_4_ compared to Ub monomers.

**Figure 3.**
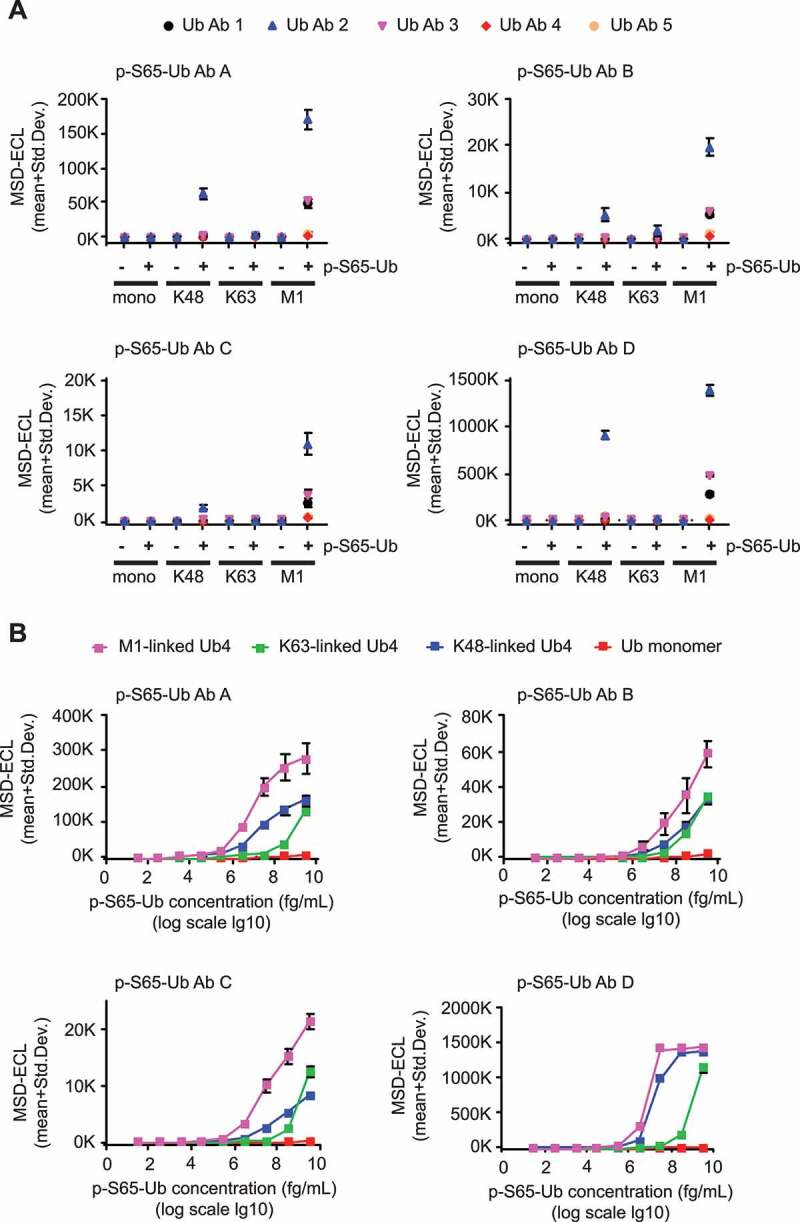
P-S65-Ub sandwich ELISAs using different Ab combinations and determination of their detection limits. (A) Rabbit p-S65-Ub Abs (AD) were used as capturing agents and combined with each of the mouse total Ub Abs (1–5) as detecting agents in identical concentrations for all Abs (1 μg/ml each). Recombinant Ub and p-S65-Ub monomers and tetramers with different chain linkage were tested in identical mass concentration. Each graph represents a different p-S65-Ub Ab combined with each of the five different total Ub Abs (1–5). Data points are shown as MSD-ECL (mean + Std. Dev) from three technical replicates. (B) Each graph represents one p-S65-Ub Ab (A-D) (1 μg/ml each) combined with total Ub Ab 2 (5 μg/ml each) for the measurement of M1-linked p-S65-Ub_4_ (pink), K48-linked p-S65-Ub_4_ (blue), K63-linked p-S65-Ub_4_ (green) and p-S65-Ub monomers in serial dilutions. Data points are shown as MSD-ECL (mean + Std. Dev) from three technical replicates with antigen concentrations graphed in log10 scale

Results are shown in individual graphs for each of the four p-S65-Ub Abs (Figure 3A). Target specificity for phosphorylated versus non-phosphorylated Ub species was given. Among different p-S65-Ub species, M1-linked tetramers resulted in highest ECL signal followed by K48- or K63-linked chains, and p-S65-Ub monomers for all Ab combinations used (Figure 3A). ECL signals were about sevenfold higher for p-S65-Ub Ab D compared to p-S65-Ub Ab A, about 50- to 100-fold higher compared to p-S65-Ub Ab B and C. Of the five total Ub Abs used, Ub Ab 2 resulted in highest ECL signal combined with each of the p-S65-Ub Abs and for all p-S65-Ub species tested. Table 2 summarizes the coefficients of variation (CVs) for the different Ab combinations used. As for the different antibody pairs tested the CVs for intraplate variability (averaged for the four recombinant p-S65-Ub species per antibody pair) were excellent for all combinations (Table 2, top row). CVs for interplate variability were at 6% for p-S65-Ub Ab D in combination with total Ub Ab 2, while all other Ab combinations were above 10%.

**Table 2.** Coefficients of variation for p-S65-Ub sandwich ELISAs

Coefficients of variation: interplate variation/intraplate variation [%]					
		p-S65-Ub Ab A	p-S65-Ub Ab B	p-S65-Ub Ab C	p-S65-Ub Ab D
1) Different Ab combinations using recombinant protein (all detecting Abs 1 µg/ml)	Ub Ab 1 Ub Ab 2 Ub Ab 3 Ub Ab 4 Ub Ab 5	34.2/4.0 34.6/3.5 38.9/2.5 37.4/1.8 63.6/2.5	32.0/1.9 30.7/1.9 42.6/3.2 52.6/1.3 49.7/1.8	27.5/2.8 25.2/2.7 32.8/3.9 36.0/3.3 53.7/3.6	38.5/5.1 6.0/4.1 47.2/3.2 21.5/3.9 32.1/3.2
2) Detecting Ab concentrations (Ub Ab 2; 5.0 µg/ml)	Ub monomers K63 chains K48 chains M1 chains	38.3/2.5 14.5/3.5 14.8/3.0 13.2/4.0	17.8/10.3 37.6/2.4 23.2/1.7 27.7/1.7	269.8/1.1 66.5/3.6 3.7/2.6 3.4/5.0	11.8/2.5 3.7/0.8 1.1/0.3 1.1/1.0
3) HEK293 cells (Ub Ab 2; 5.0 µg/ml)					5.5/2.1
4) Primary fibroblasts (Ub Ab 2; 5.0 µg/ml)					13.4/3.7
5) Mouse brain (Ub Ab 2; 5.0 µg/ml)					13.7/2.8
6) Human brain (Ub Ab 2; 5.0 µg/ml)					10.2/2.6
7) Human plasma (Ub Ab 2; 5.0 µg/ml)					5.1/4.4

We further focused on optimizing total Ub Ab 2 as detection agent by using different Ab concentrations combined with p-S65-Ub Abs A-D (Fig. S2). Increasing detecting Ab concentrations led to an increase in ECL signals and at the same time reduced differences between M1- and K48-linked p-S65-Ub_4_ species but not between p-S65-Ub tetramers and monomers (for p-S65-Ub Ab D). p-S65-Ub Ab D combined with medium (1 μg/ml) or high (5 μg/ml) concentrations of Ub Ab 2 showed highest ECL signals compared to other p-S65-Ub Abs. Interplate and intraplate CVs were determined for the four p-S65-Ub Abs each combined with total Ub Ab 2 for each of the four p-S65-Ub species tested (Table 2, row 2). Intraplate CVs were generally excellent. Interplate CVs were lowest for p-S65-Ub Ab D compared to p-S65-Ub Abs A-C at 11.8% for p-S65-Ub monomers or below 5% for poly-Ub chains. Overall, our results indicate that based on ECL signal intensity and variability the combination of p-S65-Ub Ab D and total Ub Ab 2 might represent the most promising Ab pair for a reproducible and highly sensitive sandwich ELISA.

### Determining the limit of blank, limit of detection, limit of quantification, and linear range for recombinant p-S65-Ub species

We then determined the Limit of Blank (LoB), Limit of Detection (LoD), and Limit of Quantification (LoQ) for the p-S65-Ub Ab D and total Ub Ab 2 combination, according to the EP-17 guidelines of the Clinical and Laboratory Standards Institute [40] and as discussed in *Armbruster and Pry (2008)* (Table 3) [41]. The LoB was determined as a MSD-ECL value of 251 using the formula LoB = mean _blank_ + 1.645(SD _blank_) where BSA buffer served as blank. We used serial dilutions of the different recombinant p-S65-Ub analytes (Figure 3B) to calculate the LoD = LoB + 1.645(SD _low concentration sample_) and to determine the LoQ, which is the lowest concentration of analyte that can be measured with a predefined goal for imprecision of CV under 5% for p-S65-Ub chains and under 10% for p-S65-Ub monomers. The resulting LoD values were quite similar between different pS65-Ub chains with a LoQ in the femto- to picomolar range. The LoD and LoQ of the monomeric p-S65-Ub were higher with the lowest detectable concentration in the nanomolar range (Table 3). Also the range of linearity in our assay differed between p-S65-Ub species used. For p-S65-Ub monomers, the linear range was between 390 pM and 390 nM. The linear range for K63 p-S65-Ub_4_ was between 9.7 pM and 97 nM and the linear range for both K48 p-S65-Ub_4_ and M1 p-S65-Ub_4_ was between 97 fM and 970 pM). In summary, the p-S65-Ub Ab D-total Ub Ab 2 combination detects phosphorylated tetramers in the low femto- to picomolar range and detects p-S65-Ub monomers in the low nanomolar range with excellent reproducibility.

**Table 3.** Limit of blank, limit of detection, and limit of quantification for recombinant p-S65-Ub in blocking buffer

p-S65-Ub antibody	linkage type, mono/tetramer	Limit of Blank (LoB) (MSD-ECL units)	Limit of Detection (LoD) (MSD-ECL units)	Limit of Quantification (LoQ) (CV<5%)
p-S65-Ub Ab D	Mono p-S65-Ub	251	326.3	330 ng/ml (39 nM)
	K63 p-S65-Ub_4_	251	276.6	330 pg/ml (9.7 pM)
	K48 p-S65-Ub_4_	251	256.2	3.3 pg/ml (97 fM)
	M1 p-S65-Ub_4_	251	266.1	330 fg/ml (9.7 fM)

### Cell culture-based p-S65-Ub assessment from HEK293 cells and PD patient fibroblasts

**Figure 4.**
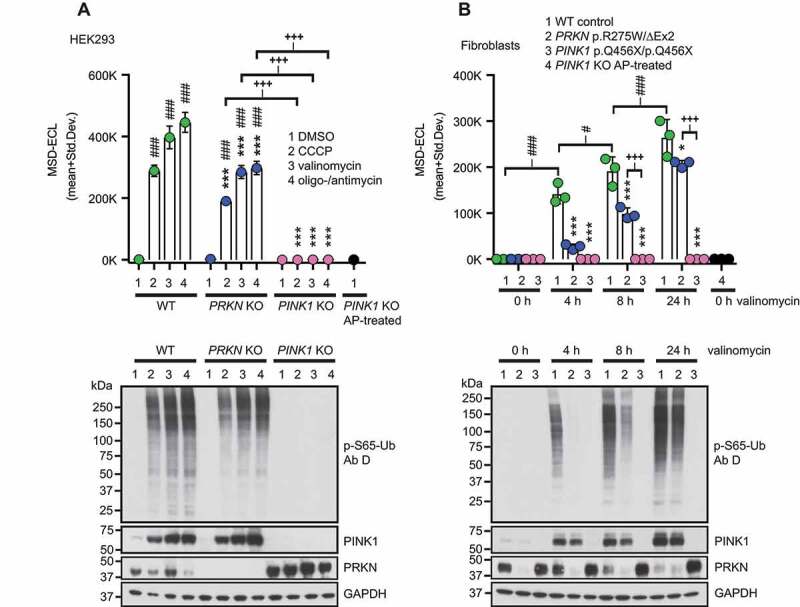
P-S65-Ub sandwich ELISA from cultured cells treated with mitochondrial depolarizers. (A) WT HEK293 cells, *PRKN* KO and *PINK1* KO HEK293 cells were treated with mitochondrial depolarizers (20 μM CCCP, 1 μM valinomycin, the combination of 10 μM oligomycin and 4 μM antimycin) or vehicle for 24 h. p-S65-Ub levels were determined by sandwich ELISA and representative western blots are shown below the graph. (B) PD patient-derived human skin fibroblasts carrying no mutation (WT), compound heterozygous *PRKN* mutations p.R275W/ΔExon2 or homozygous *PINK1* p.Q456X mutations were treated with 1 μM valinomycin for 0, 4, 8, or 24 h. *PINK1* KO HEK293 cells treated with AP were used as additional negative control. p-S65-Ub levels were determined by sandwich ELISA and representative western blots are shown below the graph. MSD-ECL values (mean + Std. Dev.) are shown from three replicates for both p-S65-Ub ELISAs in A and B. Two-Way ANOVA and Tukey’s post-hoc test (* p < 0.05, ** p = 0.005, *** p < 0.0001). Asterisks (*) indicate the comparison to WT samples for the same treatment (A) or time point (B). Number signs (#) indicate the comparison to the control treatment within the same genotype. Plus signs (+) indicate comparison between *PRKN* and *PINK1* mutant genotypes

We next attempted to use the optimized sandwich ELISA with p-S65-Ub Ab D and total Ab 2 to measure p-S65-Ub levels in lysates from parental wild type (WT) as well as *PRKN* or *PINK1* knockout (KO) HEK293 cells treated with mitochondrial uncoupling agents compared to vehicle control. Treatment of WT and *PRKN* KO with CCCP, valinomycin, or oligomycin-antimycin for 24 h resulted in significant increases in p-S65-Ub ECL signal compared to DMSO-treated controls (Figure 4A**, top)**. While WT HEK293 cells showed significantly higher p-S65-Ub ECL signal compared to *PRKN* KOs for the same treatments, treated *PINK1* KOs had only signal at background level (p < 0.0001 for all comparisons). We used alkaline phosphatase (AP)-treated samples as additional negative controls, which resulted in p-S65-Ub levels that were similar to *PINK1* KO HEK293 cells irrespective of their treatment. The CVs for p-S65-Ub sandwich ELISAs in HEK293 cells were 5.5% for interplate and 2.1% for intraplate variation. The absence of PINK1 or PRKN in the respective genome-edited HEK293 KO cells was validated by western blot (Figure 4A**, bottom**). Likewise, increased p-S65-Ub levels were confirmed and were accompanied by elevated PINK1 levels upon treatment with the different mitochondrial stressors.

In addition to mitochondrial stress, we tested whether blocking autophagic-lysosomal degradation in WT cells using bafilomycin A_1_-treatment would cause a detectable increase in p-S65-Ub levels in our sandwich ELISA, using both *PINK1* KO and vehicle-treated WT cells as controls. Importantly, p-S65-Ub levels were significantly increased in bafilomycin A_1_-treated WT cells compared to DMSO-treated WT cells and to *PINK1* KO cells (p < 0.0001 for all comparisons) (Fig. S3). Of note, using this assay we were even able to detect significant differences between DMSO-treated WT and *PINK1* KO HEK293 cells (p = 0.0001) in the absence of autophagy inhibitors.

To confirm findings in primary cells from PD patients, we used skin fibroblasts and assessed p-S65-Ub levels in carriers of homozygous *PINK1* (p.Q456X) or compound heterozygous *PRKN* (p.R275W/ΔEx2) mutations relative to healthy controls. Among WT fibroblast samples, the assay showed a significant increase in p-S65-Ub levels over time with valinomycin treatment compared to untreated WT fibroblasts (0 h) (p < 0.0001 for all comparisons) (Figure 4B, top). Similarly, treated *PRKN* mutant fibroblasts showed an overall significant increase over time compared to untreated cells (0 h), though only at later time points (p < 0.0001 at 8 h and 24 h). Upon stress, *PRKN* mutant cells had significantly lower p-S65-Ub accumulation compared to WT within individual time points (p < 0.0001 for 4 h and 8 h; p = 0.01 for 24 h), while the signal in *PINK1* mutant fibroblasts was similar to buffer blanks. The CVs for p-S65-Ub sandwich ELISAs in human fibroblasts were 13.4% for interplate and 3.7% for intraplate variation. The corresponding pS65-Ub levels in WT control cell and *PINK1* or *PRKN* mutant fibroblast samples were validated by western blot (Figure 4B, bottom).

In summary, the sandwich ELISA was able to distinguish between all three genotypes when comparing WT controls to *PINK1* or *PRKN* mutant cells. Lack of detectable p-S65-Ub signal in *PINK1* mutant cells confirmed the specificity of the antibodies. Moreover, the MSD assay was sensitive enough to not only identify reduced p-S65-Ub levels in *PRKN* mutant cells upon stress, but more importantly, also baseline levels of the mitophagy tag in WT controls in the absence of stress.

### P-S65-Ub detection in mouse brains, human autopsy brain samples, and blood plasma

**Figure 5.**
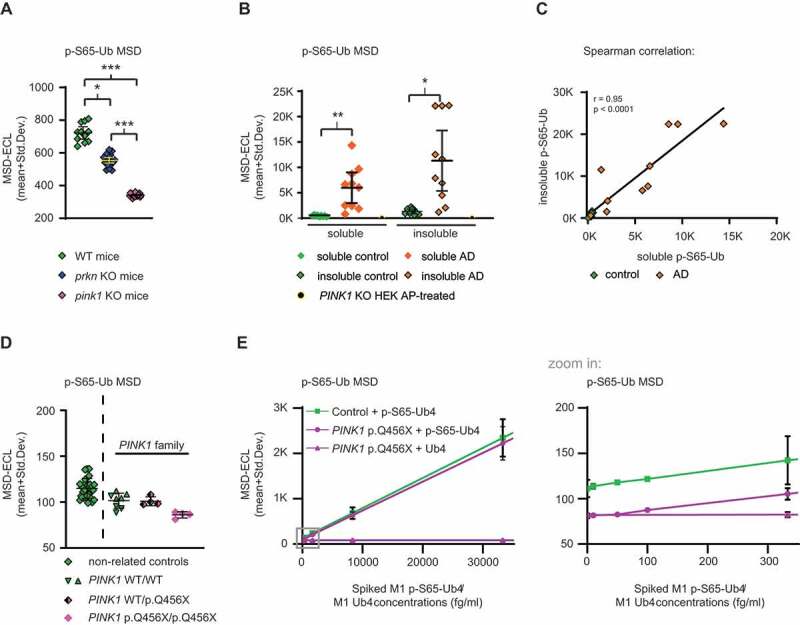
P-S65-Ub sandwich ELISA from mouse brain, human autopsy frontal cortex and human plasma. (A) Mouse brains lysates from WT (n = 11), *prkn* KO (n = 20), and *pink1* KO (n = 19) mice were analyzed for p-S65-Ub levels by ELISA. Data points are shown as MSD-ECL (mean + Std. Dev.). No difference in sex was observed for WT mice, *pink1* KO, or *prkn* KO mice. Kruskal-Wallis test combined with Dunn’s multiple comparison test (* p < 0.05, *** p < 0.0001). (B) Frontal cortex from age- and sex- matched AD (n = 10) vs. neurologically normal control cases (n = 9) were analyzed for p-S65-Ub levels in soluble and insoluble fractions by sandwich ELISA. *PINK1* KO HEK293 cells treated with AP were used as negative control. Kruskal-Wallis test followed by Dunn’s multiple comparison test (* p < 0.05, ** p < 0.005). (C) Spearman correlation with linear regression for p-S65-Ub in insoluble vs. soluble fraction for AD (orange diamonds) and control cases (green diamonds). (D) Human plasma p-S65-Ub levels were determined in blood samples from non-related controls (n = 29) as well as from control family members without *PINK1* mutations (n = 2) and related individuals carrying a heterozygous (n = 1) or homozygous (n = 1) pathogenic *PINK1* mutation (p.Q456X). Non-related controls are separated by a dashed line with each symbol reflecting a single run for each individual. The raw data for the PINK1 family is shown as symbols per run for each sample. (E) Spike-recovery of phosphorylated M1 p-S65-Ub_4_ and non-phosphorylated M1 Ub_4_ into *PINK1* p.Q456X/p.Q456X and control plasma. The gray boxed region of the graph (left side) covering low spiked M1 p-S65-Ub_4_ respective total M1 Ub_4_ concentrations is magnified in the graph on the right side (zoom in)

Next, we aimed to measure p-S65-Ub levels with our best antibody pair in mouse hemi brain lysates from a cohort of *pink1* KO (n = 19), *prkn* KO (n = 20), and WT mice (n = 11) under non-stimulated conditions as a proxy for baseline mitophagy levels. Since there was no detectable sex difference (data not shown), the results for male and female mice were combined. p-S65-Ub levels were significantly different between all three groups with highest ECL signals in WT mice followed by *prkn* KO mice and then *pink1* KO mice (Figure 5A). The CVs for the mouse brain analysis using p-S65-Ub Ab D were 13.7% for interplate variation and 2.8% for intraplate variation. Importantly, results demonstrated that our p-S65-Ub MSD assay was able to detect PINK1-PRKN-mediated mitophagy in mouse brains at baseline with significant differences between WT, *prkn* KO, and *pink1* KO mice, similar to the cell culture experiments.

An essential question to be addressed was whether our p-S65-Ub MSD assay is applicable for testing mitophagy changes in human tissue. Therefore, we attempted to assess p-S65-Ub levels in autopsy frontal cortex from age- and sex-matched neurologically normal controls (n = 9) and AD cases (n = 10). Each case was biochemically fractionated into a soluble (RIPA) and insoluble (RIPA+1% SDS) fraction. p-S65-Ub MSD-ECL values were significantly increased in AD cases compared to controls in both soluble and insoluble fractions (Figure 5B). The CVs for the human brain analysis using p-S65-Ub Ab D were 10.2% for interplate variation and 2.6% for either intraplate variation. The CV for interplate variation was averaged and can be subdivided into a CV soluble fraction (5.5%) and a CV insoluble fraction (14.8%), indicating less assay variation in analyzing the soluble compared to the insoluble fraction. A Spearman correlation indicates that soluble and insoluble p-S65-Ub levels are significantly associated with each other (Figure 5C), suggesting that either fraction can be used for this analysis.

Last, we aimed to determine whether p-S65-Ub is present and detectable in human blood plasma. We received freshly collected samples from four related family members with or without a rare loss-of-function mutation in *PINK1* [42]. These samples included plasma from all three *PINK1* genotypes – WT/WT controls (n = 2), WT/p.Q456X (n = 1) and p.Q456X/p.Q456X (n = 1). Consistent with our previous report [43] and data from fibroblasts included herein, the p-S65-Ub ELISA showed the lowest raw values for the sample from the symptomatic, homozygous p.Q456X carrier compared to the plasma from the unaffected heterozygous and WT controls. While the sample number is certainly too low to statistically analyze the effect, confidence in the detection of p-S65-Ub was strengthened by the finding that 29 additional samples from unrelated controls [44] showed very little spread and every sample had higher p-S65-Ub values than the homozygous *PINK1* p.Q456X mutation carrier (Figure 5D). The CVs for interplate (5.1%) and intraplate (4.4%) variation were excellent for blood plasma p-S65-Ub assessment. To further corroborate the presence and detectability of p-S65-Ub in plasma and to estimate the matrix effect, we performed spike-and-recovery experiments. Spike-in of serially diluted recombinant M1 p-S65-Ub_4_ or non-phosphorylated M1 Ub_4_ chains into either homozygous *PINK1* p.Q456X plasma or control plasma revealed differences even at lowest, identical spiked concentrations between these plasma samples (Figure 5E). Furthermore, non-phosphorylated M1 Ub_4_ was not detected in plasma using our p-S65-Ub ELISA even when spiked in at high nanomolar concentrations (Figure 5E), confirming that the assay does not cross-react with non-phosphorylated Ub present in plasma. The LoB in plasma was determined as 85 MSD-ECL units, and the LoD for M1 pS65-Ub_4_ was 88.1 MSD-ECL units. The LoQ for M1 pS65-Ub_4_ was calculated as 100 fg/ml (2.9 fM). The CVs for p-S65-Ub spike-and-recovery experiments into plasma were 8.5% for interplate and 2.3% for intraplate variation.

In summary, results from human specimens demonstrated that p-S65-Ub levels are not only detectable in postmortem frontal cortex by sandwich ELISA, but are significantly different in both soluble and insoluble fractions in AD vs. control. Together with our immunohistochemical findings this strongly underscores the involvement of aberrant mitophagy flux in neurodegenerative diseases such as AD. Moreover, p-S65-Ub is detectable in human blood plasma with our MSD assay and should be explored as a disease marker in further clinical samples.

## Discussion

Here we developed a novel protocol and established a highly sensitive method to measure p-S65-Ub as a readout for PINK1- and PRKN-dependent mitophagy by sandwich ELISA on a MSD platform. For this, we screened and validated several antibodies using recombinant proteins, genome-edited and primary patient cells, mouse and human autopsy brain tissues as well as human plasma samples. We corroborated the selectivity and specificity of the various Abs and combinations thereof in different applications and using multiple controls. In contrast to immunostaining of cells or tissues or other biochemical readouts like western blot, the MSD assay was able to detect even extremely low p-S65-Ub levels that occur at baseline in unstressed human cells and mouse brain tissues. Noteworthy, the assay is sensitive enough to discriminate between samples of all three genotypes in human cells and mouse brain (WT controls vs. loss of PINK1 or PRKN function), to differentiate pathological conditions in human autopsy brain tissue (neurologically normal controls vs. AD), and even to detect p-S65-Ub in human blood.

First, we set out to characterize the various Abs against phosphorylated (p-S65) or total Ub *in vitro* using recombinant monomeric and poly-Ub chains of defined concentrations, lengths, and linkages. p-S65-Ub tetramers were generally detected with at least >150-fold higher ECL signal intensity compared to a 4-fold molar excess of p-S65-Ub monomers. This is likely caused by steric hindrance between two large Abs (~150 kDa) targeting a single, small Ub unit (8.6 kDa) in a sandwich-type format. In contrast, in a Ub chain, such as the tetramer, both antibodies might bind to different moieties and therefore result in increased sensitivity. However, we also detected stark differences when comparing poly-Ub chains of different linkage types. This may be caused by the close proximity of serine 65 and the branching lysine residue (e.g. K63) or by overlapping epitopes of Abs targeting phosphorylated or total Ub. Alternatively, this may also relate to p-S65-Ub adopting a so-called “invisible conformation” through retraction of the C-terminal tail [45]. In a poly-Ub chain, this would likely result in a different overall topology that could also affect recognition by some of the Abs. While it is of note that all Ub Abs tested targeted their antigens dependent on chain length and linkage, our screening on both western blot and MSD resulted in the identification of an Ab pair that detects phosphorylated Ub monomers in the low nanomolar range and Ub tetramers in the low femto- to picomolar range with excellent reproducibility.

Second, as biological samples, we used CRISPR-Cas9 genome-edited cells in which we had deleted *PINK1* or *PRKN* as well as primary skin fibroblasts obtained from PD patients carrying loss-of-function mutations in either gene. While almost any Ab and method was able to detect the very high levels of p-S65-Ub upon mitochondrial depolarization, only our new MSD assay captured the extremely low baseline levels in WT cells. In addition, p-S65-Ub levels were slightly elevated upon inhibition of lysosome function, which leads to the accumulation of p-S65-Ub that otherwise would have already been turned over (at the time of the experiment). The delta in p-S65-Ub levels when comparing WT controls side-by-side with cells with loss of PINK1 function or dephosphorylated samples likely reflects the basal cellular PINK1-PRKN-dependent mitophagy rates. However, upon mitochondrial damage, the MSD assay was also able to discriminate between WT controls and cells with loss of PRKN function, which is activated and recruited by p-S65-Ub upon stress and then swiftly amplifies the mitophagy signal. Given this feedforward loop between the Ub kinase PINK1 and the Ub ligase PRKN, our p-S65-Ub MSD assay can be used as a readout for either enzymatic function.

Third, we investigated p-S65-Ub levels in mouse hemibrain lysates as more complex biological samples. It is noteworthy that neither *pink1* nor *prkn* KO mice present overt neurodegenerative phenotypes [46–49], at least not under basal conditions. Old age, exhaustive exercise, high-fat diet, exposure to pathogens and toxins or other disease conditions may challenge animals sufficiently to trigger the wider activation of the PINK1-PRKN mitophagy pathway and as such may reveal phenotypes of such sensitized genetic background [47,50–52]. While immunohistochemistry was not sensitive enough to detect p-S65-Ub in mouse brain at unstimulated conditions, the MSD assay was able to capture signal in these samples. Perhaps even more striking, the p-S65-Ub ELISA allowed discrimination between all three genotypes – WT controls, *pink1* KO and *prkn* KO. This is in contrast to cells in culture, where additional exogenous mitochondrial stress was required to reveal significantly different p-S65-Ub levels in WT controls compared to cells with loss of PRKN function. Importantly, a recent report suggested that very old *prkn* KO mice (>110 weeks of age) start to show PD-like symptoms resulting from dopaminergic neuron death that is related to mitophagy defects [50]. However, using our p-S65-Ub MSD assay we here identify a much earlier detectable “biochemical defect” in a relatively young cohort of mice (mean ages between 15–33 weeks).

Fourth, we tested the ability of our MSD assay to quantify p-S65-Ub in human pathological and clinical samples. Toward this, we used autopsy brain material isolated from frontal cortex of AD patients and neurologically normal cases. The sandwich ELISA was able to discriminate between diseased and control samples and detected significantly increased levels of p-S65-Ub in both the soluble and insoluble fraction of AD frontal cortex. We previously had used immunohistochemistry to identify changes in p-S65-Ub levels in autopsy brain with increased age or Lewy body disease and more recently also in AD brain tissue [34–36,53]. As expected based on our data using immunohistochemistry, p-S65-Ub levels were elevated over neurologically normal controls in both sporadic forms of AD and Lewy body disease, which is indicative of an increased pool of dysfunctional mitochondria and/or impaired autophagic flux. This is in contrast to the sharp decrease in p-S65-Ub that is expected and observed in cell and autopsy brain tissue from rare cases with pathogenic *PRKN* or *PINK1* loss-of-function mutations [34,36,43,54], which inhibit or even abrogate the initiation of mitophagy. While it remains to be determined why exactly p-S65-Ub levels are increased in cases with sporadic disease, efforts are underway assessing the levels of the mitophagy tag in the context of different neuropathologies across a spectrum of diseases. In contrast to the fairly high levels in human autopsy brain, in plasma our MSD assay picked up very low p-S65-Ub signals in samples from controls that however were consistently higher than the signal measured in a homozygous loss-of-function *PINK1* plasma sample. Given its cellular origin, p-S65-Ub is either actively secreted, passively diffuses, or is released as the result of cell death and ultimately presents extracellularly in the plasma. Although we currently do not know the exact source, plasma concentrations of non-phosphorylated Ub have been found elevated in various diseases in the high picomolar to nanomolar range [55–58]. Here we show that p-S65-Ub is present and detectable and estimate that the absolute amount of in plasma as detected in our ELISA is in the femtomolar range. While it remains uncertain whether p-S65-Ub levels can be used as a diagnostic or prognostic disease marker in blood, given the sensitivity of the MSD assay, it should at least be possible to discriminate true loss-of-function PINK1 or PRKN patients from subjects harboring single heterozygous mutations or more ambiguous variants of unknown significance.

Though highly sensitive, one important limitation of our p-S65-Ub MSD assay is the current inability to absolutely quantify the mitophagy tag. Given the uncertain composition of the p-S65-Ub signal in biological specimens with potentially mixtures of poly-Ub chains of different lengths and linkages, it is simply not possible to determine absolute quantities. However, we determined the respective LoD and LoQ in human plasma and blocking buffer as well as the linear range using recombinant Ub species to approximate the values for complex biological samples. A few recent reports collectively suggest that the majority of the Ub signal on mitochondria may stem from mono-ubiquitination of substrate proteins or short poly-Ub chains and that only the most distal Ub in a chain might be phosphorylated at serine 65 [59–61]. Regardless, also technical advances in the field may help untangle this complexity going forward. For instance, an additional processing step with a deubiquitinating enzyme to cleave Ub off substrate proteins and to digest more complex chains into Ub monomers may facilitate absolute quantification of p-S65-Ub. While we found that detection of monomeric p-S65-Ub was less sensitive in the current sandwich ELISA, future nanobodies or other smaller, recombinant binders may allow targeting individual p-S65-Ub moieties with high sensitivity.

In the meantime, relative quantification as done herein, requires establishing the baseline levels of p-S65-Ub by comparing WT controls side-by-side with PINK1 deficient or dephosphorylated samples. While this might be less important for extreme stress conditions in cells or certain pathological samples, this will be key in specimens with low p-S65-Ub such as physiological conditions or in clinical samples such as human blood. It is important to note that p-S65-Ub is a transient, dynamic marker and this might contribute to its low levels at baseline. While p-S65-Ub acts as an allosteric activator of and receptor for PRKN it also serves as the mitophagy tag and as such is turned over with removal of damaged mitochondria. Anything below that baseline is likely due to reduced expression or impaired PINK1 and/or PRKN activity while higher levels are due to either increased mitochondrial stress and/or decreased autophagic flux as seen with stress, age, and disease. Regardless, both are indicative of altered mitophagy. As such, levels of the mitophagy tag p-S65-Ub are an early, robust indicator of damage and should be further explored as a potential disease and pharmacodynamics biomarker.

## Materials and methods

### Ethics approval

All brain samples are from autopsies performed after approval by the legal next-of-kin. Research on de-identified postmortem brain tissue is considered exempt from human subjects’ regulations by the Mayo Clinic Institutional Review Board. Human blood plasma and primary dermal fibroblast collection, processing, and analyses were approved by the Institutional Review Boards of Mayo Clinic and the Katowice Medical University of Silesia, Katowice, Poland. All procedures involving animals were in accordance with the ethical standards established by Mayo Clinic and approved by the Institutional Animal Care and Use Committee at University of Alabama.

### Recombinant proteins

All used recombinant protein were purchased from Boston Biochem: Ub monomer (U100H), p-S65-Ub monomer (U-102), K48 Ub_4_ (UC-210b), K48 p-S65-Ub_4_ (UC-250), K63 Ub_4_ (UC-310b), K63 p-S65-Ub_4_ (UC-350), M1 Ub_4_ (UC-710b), M1 p-S65-Ub_4_ (UC-750), p-S65-PRKN (E3-166).

Recombinant proteins were diluted in RIPA buffer (50 mM Tris, pH 8.0 [affymetrix, 75,825], 150 mM NaCl [Fisher Scientific, BP-358], 0.1% SDS [Bio-Rad, 1,610,302], 0.5% deoxycholate [Sigma, D6750], 1% NP-40/Igepal [United States Biochemical, 19,628]). In western blots 20 ng of protein was loaded per well for all Ub and p-S65-Ub monomers and tetramers and 30 ng per well loaded for p-S65-PRKN. Please note the use of 4-fold molar excess for Ub and p-S65-Ub monomers relative to Ub and p-S65-Ub tetramers to accommodate for equal numbers of epitopes per load.

### Cell culture and treatment

Primary human dermal fibroblasts collected from *PINK1* or *PRKN* mutation carrier and control fibroblasts (cryopreserved HDF cells; [Cell Applications Inc., 106–05A]) were grown in Dulbecco’s modified Eagle medium (DMEM [Thermo, 11,965,118]) supplemented with 10% fetal bovine serum (FBS [Neuromics, FBS001800112]), 1% PenStrep (Thermo, 15,140,122) and 1% non-essential amino acids (Thermo, 11,140,050). Human embryonic kidney cells 293E (HEK293E [ATCC, CRL-3216]) were cultured in DMEM supplemented with 10% FBS. All cells were grown at 37°C, 5% CO_2_:air in humidified atmosphere. *PINK1* or *PRKN* KO cells were generated by co-transfecting cells with *CAS9* cDNA [62] and guide RNAs targeting *PINK1* exon 6 (TACGTGGATCGGGGCGGAAA) or *PRKN* exon 7 (GTGTGACAAGACTCAATGAT) using XtremeGene 9 (Sigma, 6,365,787,001). pX330-U6-Chimeric_BB-CBh-hSpCas9 was a gift from Feng Zhang (Addgene, 42,230). Cells were plated as single cells in 96-well plates and expanded cell clones were screened for the absence of PINK1 or PRKN protein, respectively, by western blot. Positive cell clones were validated by Sanger sequencing of the target and of potential off-target sites.

After treatment with mitochondrial depolarizers CCCP (Sigma, C2759), valinomycin (Enzo Life Science, BML-KC140-0025), oligomycin A (Sigma, 75,351) + antimycin (Sigma, A8674), autophagic-lysosomal inhibitor bafilomycin A_1_ (LC Laboratories, B1080) or vehicle DMSO (Sigma, D4540), cells were lysed in RIPA buffer containing protease inhibitor cocktail and phosphatase inhibitors (Sigma-Aldrich, 11,697,498,001 and 04906837001). Cell lysates were cleared for 10 min, 4ºC at 20,817 x g and protein concentrations were determined by BCA assay (Thermo Fisher, 23,225).

### Western blotting and silver staining

Protein electrophoresis was performed at room temperature (22ºC) in electrophoresis running buffer (192 mM glycine [Gold Biotechnology, G-630], 25 mM Tris, pH 8.3, 0.1% SDS). Recombinant proteins were separated at 100 V using a Mini-PROTEAN electrophoresis cell (Bio-Rad) with 4–20% gradient polyacrylamide gels (Bio-Rad, 4,561,094). Cell lysates were diluted in Laemmli buffer (62.5 mM Tris, pH 6.8, 1.5% SDS, 8.33% glycerol [Fisher Scientific, BP2291], 1.5% β-mercaptoethanol [Sigma, M3148], 0.005% bromophenol blue [Sigma, B5525]) and subjected to SDS-PAGE using 8–16% Tris-glycine gels (Invitrogen, EC60485BOX). Proteins in polyacrylamide gels were subsequently silver stained with Pierce Silver stain Kit (ThermoFisher, 24,612) or transferred on polyvinylidene fluoride membranes (Millipore, Immobilon PSQ, ISEQ00010 or PVH00010) at 4ºC, 100 V in transfer buffer (192 mM glycine, 25 mM Tris, pH 8.3, 20% methanol [Pharmcoaaper, 339000ACSCSGL]) using either a Mini or a Criterion Trans Blot Cell (Bio-Rad). Polyvinylidene fluoride membranes containing recombinant proteins were post-fixed immediately after transfer in 4% (w:v) paraformaldehyde (Sigma, 441,244) in PBS (137 mM NaCl, 2.7 mM KCl [Sigma, P3911], 10 mM Na_2_HPO_4_ [Sigma, S5136], 1.8 mM KH_2_PO_4_ [Sigma, P5655], pH 7.4) for 0.5 h at 22ºC, washed twice with TBS-T (50 mM Tris, pH 7.4, 150 mM NaCl, 0.1% Tween-20 [Sigma, P1379]) before membranes were blocked for 1 h in 5% dry milk powder (Thermo Scientific, OXLP0031B) in TBS-T on a shaking platform under gentle agitation. Primary Abs were incubated overnight in 5% BSA (Boston BioProducts, P-753) in TBS-T at 4ºC (Table 1), and membranes were washed 3 times with TBS-T before secondary Ab addition and incubation for 1 h at room temperature. Proteins were visualized after washing in TBS-T using Immobilon Western Chemiluminescent HRP Substrate (Millipore Sigma, WBKLS0500) on Pro Signal Blotting film (Genesee Scientific, 30–810 L).

Quantification of western blots and silver stained gels was performed using *Image Studio Lite* software. Intensity levels per area measured for protein bands from western blots were background subtracted and then normalized to the intensity levels of protein bands (from identical proteins) measured in silver stained gels.

### Immunofluorescence staining of cells and high-content imaging

To quantify p-S65-Ub levels, automated high-content imaging was employed as recently described [43,63]. In brief, cells were seeded in 96-well imaging plates (Fisher Scientific, 08772225) and allowed to attach overnight. Cells were then treated for 0 or 24 h with 1 µM valinomycin. Cells were washed once in PBS (Boston BioProducts, BM-220), fixed for 10 min in 4% paraformaldehyde (Sigma-Aldrich, 441,244) and permeabilized with 1% Triton X-100 (Fisher Scientific, BP151) in PBS. Cells were stained using different p-S65-Ub Abs (see dilution details in the table) and with Hoechst 33,342 (Invitrogen, H21492l; 1:5000). Plates were imaged on a BD Pathway 855 (BD Biosciences, San Jose, CA, USA) with a 20x objective using a 2 × 2 montage (no gaps) with laser autofocus every second frame. Raw images were processed using the built-in AttoVision V1.6 software. Regions of interest were defined as nucleus and cytoplasm using the built-in “RING-2 outputs” segmentation for the Hoechst channel after applying a shading algorithm. Background signal at 0 h was subtracted for each Ab and values were normalized to the highest value.

### P-S65-Ub sandwich ELISA

To establish the best combination of antibodies for the sandwich ELISA all four rabbit p-S65-Ub Abs (#A-D) were used as capturing agents and combined with each of the five different total mouse Ub Abs (#1-5) as detecting tools (Table 1). Capturing Abs were used in a concentration of 1 μg/ml in 200 mM sodium carbonate buffer pH 9.7 and coated overnight at 4ºC with 30 μl per well in 96-well MSD plate (MSD, MULTI-ARRAY® 96-well Plate; L15XA-3). The next morning MSD plates were washed 3 times with 0.22-micron filtered ELISA blocking buffer (150 mM Tris, pH 7.4, 150 mM NaCl, 0.1% [v:v] Tween-20, 1% BSA [w:v]) using a 1200 μl multi-(12)-channel pipettor with 300 μl per well and incubated for 1 h at 22ºC without shaking. Washing was performed by plate inversion and gentle tapping on paper towels (not by pipette aspiration) and with no incubation time between washes. All samples were run in duplicates and diluted in blocking buffer. Initial experiments using recombinant protein (Figure 3A) were performed using a single protein concentration of 33 ng/ml corresponding to 3.88 nM for Ub and p-S65-Ub monomers respective 968 pM for Ub and p-S65-Ub tetramers. In order to determine the sensitivity of the different p-S65-Ub Abs each combined with mouse Ub Ab 2, recombinant antigens were further analyzed as 10-fold serial dilutions. Samples were added to the plate using 100 ng – 1 fg per 30 μl total volume for each well (resulting in a final concentration range of 388 nM – 3.88 fM for monomers and 96.8 nM – 968 aM for tetramers).

For the quantification of pS65-Ub in HEK293 cells or fibroblasts, 5–10 µg of cell lysate was used per well. Detection of pS65-Ub in mouse or human brain was performed using 30 µg of lysate per well. For the p-S65-Ub ELISA in human plasma we used neat (undiluted) samples in quadruplicates (30 μl per well) in four independent runs. Antigens were incubated for 2 h at 22ºC on a microplate mixer (USA Scientific, 8182–2019) at 500 rpm and three washing steps were then performed as described before. Detecting mouse Abs (Ub 1–5, Table 1, 1 µg/ml) were added in blocking buffer in 30 μl total volume per well. In experiments with cell lysates, brain lysates or plasma, detecting Ub Ab 2 was used at a concentration of 5 μg/ml. *PINK1* KO HEK293 cells were used as additional negative control and bafilomycin A_1_-treated WT HEK293 cells (400 μM, 24 h) were used as positive control. Detecting antibodies were incubated for 2 h at 22ºC on a microplate mixer at 500 rpm. After three washing steps, SULFO-TAG labeled goat anti-mouse Ab (MSD, R32AC-1) was next added in blocking buffer in a dilution of 1:500 using 50 μl per well and incubated for 1 h at 22ºC on a microplate mixer at 500 rpm. After another three washing steps, 150 μl MSD GOLD Read Buffer (MSD, R92TG-2) were finally added to each well and the plate being read on a MESO QuickPlex SQ 120 reader.

### Mouse brain

*prkn* KO and *pink1* KO mice were generated under a C57BL/6 background as described previously [48,49]. Mice were anesthetized and intracardial perfused with cold PBS (4ºC) and brains were removed and immediately flash-frozen in liquid nitrogen. A total of 50 mouse hemi brains were received from Dr. Matthew Goldberg (University of Alabama, USA) and included 11 WT mice (6 female, 5 male), 20 *prkn* KO mice (8 female, 12 male), and 19 *pink1* KO mice (10 female, 9 male). Average mouse age per genotype (combined sexes) was (mean ± Std.Dev.): WT: 105.5 ± 29.7 days; *prkn* KO: 233.4 ± 120.7 days; *pink1* KO: 142.1 ± 17.1 days. Mice with identical genotype originated either from the same or different litters. Mice with different genotype were not littermates.

Frozen brains were stored at −80ºC and kept on dry ice and completely frozen during handling including weighing. Throughout all tissue lysis/homogenization steps, mouse brain tissues/lysates were kept at 4ºC. Mouse brain tissue was first homogenized in 5 volumes (relative to brain weight e.g. 100 mg brain tissue in 500 μl) of ice-cold TBS supplemented with protease and phosphatase inhibitors. Brains were homogenized first by passing through a 1-ml pipette tip followed by 10 strokes up and down through a 27xg needle. Brain homogenates were divided in 100 μl aliquots, flash-frozen in liquid nitrogen, and stored at −80ºC. Tissue lysis was completed by adding 25 μl of 5x RIPA buffer to 100 μl of mouse brain homogenate on ice, mixed, passed through a 27xg needle (10 times up and 10 times down) and incubated at 4ºC for 30 min. Insoluble material, lipids, and nucleic acids were removed by serial centrifugation (2x) at 20,000 x g, 4ºC for 10 min.

### Human autopsy brain

Demographic information: Autopsied human brain specimens originate from frontal cortex in AD vs. neurologically normal controls. As for the control cohort (n = 9) the average age was 84.0 ± 6.2 years (mean ± Std.Dev.); 44% females; Braak tangle stage was between I–III (5x I; 3x II, 1x III); Thal phase was determined in 4 out of 9 control cases (3x 0, 1 × 2). In the AD cohort (n = 10) the average age was 84.4 ± 3.3 years (mean ± Std.Dev.); 50% females; Braak tangle stage was between V–VI (6x V, 4x VI); Thal phase was determined in 5 out of 10 AD cases (4x 5, 1 × 3).

Human brain tissue was stored at −80ºC and kept on dry ice and completely frozen during handling including weighing. Frozen human brain chips from frontal cortex were lysed in five volumes of RIPA buffer supplemented with protease and phosphatase inhibitors. Tissue lysis/homogenization was performed at 4ºC during all steps by triturating first through a 1 ml pipette tip followed by 10 strokes up and down through a 23xg needle and finally 10 times through a 27xg needle and incubated for additional 30 min at 4ºC. Insoluble material, lipids, and nucleic acids were removed by centrifugation at 20,000 x g, 4ºC for 10 min. The resulting fraction was termed RIPA fraction (soluble fraction). The pellet was washed twice with RIPA buffer to remove remaining RIPA-soluble material and the remaining pellet was heated for 5 min at 95ºC in RIPA buffer supplemented with 1% SDS final concentration. Volumes used for this RIPA+SDS fraction (insoluble fraction) were identical to corresponding volumes for each RIPA fraction. Pellets from the RIPA+SDS (insoluble fraction) fraction were washed twice in RIPA with 1% SDS and subsequently solubilized in 8 M urea (Oakwood Chemical, 044699) with 4% SDS, run on western blots, probed for housekeeping proteins, and did not contain any detectable protein levels.

### Immunohistochemistry of human postmortem brain

Hippocampal sections from paraffin embedded postmortem AD brain tissue were cut at a thickness of 5 microns and allowed to dry overnight in a 60°C oven. Following de-paraffinization and rehydration, target retrieval was performed by steaming the sections for 30 min in deionized water. Immunostaining was performed with a Dako Autostainer using Envision Plus kit (Agilent, K4011). Endogenous peroxidase was blocked for 5 min with 0.03% hydrogen peroxide. Sections were then treated with 5% normal goat serum (Invitrogen, 16,210,072) for 20 min. Subsequently, sections were incubated for 45 min at room temperature in different dilutions of primary Abs against p-S65-Ub (Ab A-D). After incubation with primary Ab, sections were incubated in Envision-Plus rabbit- or mouse-labeled polymer HRP (Agilent, K4011) for 30 min at room temperature. Peroxidase labeling was visualized with the chromogen solution 3, 3ʹ-diaminobenzidine. The sections were then counterstained with Lerner 1-hematoxylin (Fisher Scientific, CS400-1D) and cover slipped with Cytoseal mounting medium (Thermo Scientific, 8310). After drying, all sections were scanned with an Aperio AT2 digital pathology scanner (Leica Biosystems, Wetzlar, Germany).

### Human plasma preparation and analysis

Control plasma samples were obtained from the Harvard Biomarkers Study (https://www.bwhparkinsoncenter.org) [44] with the following basic demographics: n = 29; sex: 11 F, 18 M; age: 63.1 ± 10.2 years (mean ± Std.Dev.). Additional samples were obtained from four related family members with or without a rare, pathogenic *PINK1* mutation [42]: WT controls (n = 2; both M; age; 29 and 31), p.Q456X heterozygote (n = 1; F; age 52), and p.Q456X homozygote (n = 1; M; age 46). These blood samples were processed within 30 min of collection. In brief, blood (10 ml) was collected in EDTA vacutainers; tubes were inverted to mix and subsequently kept on ice. Samples were centrifuged for 15 min at 4ºC at 2,465 x g. Supernatants were aliquoted on ice with 500 μl per tube, frozen on dry-ice. For p-S65-Ub ELISAs, samples were thawed on ice, centrifuged for 10 min, 4ºC at 20,000 x g, aliquoted into 100 μl and directly used as needed or flash-frozen in liquid nitrogen.

### Statistical analysis

Each sample was run in duplets on the same plate with at least three replicates. For the intraplate CV, mean and standard deviation were calculated for each sample of the specific cohort that was run in duplicates on each plate. The mean was then calculated from all intraplate CVs for a specific cohort and antibody combination and shown in Table 2. As for the interplate CV, the mean from each duplicate run of a specific sample and each subsequent technical repeat was used to calculate the overall mean (i.e. the mean of the means). Interplate CVs for specific samples and conditions were then used to calculate cohort-specific interplate CVs (e.g. for all K48 p-S65-Ub_4_) which are shown in Table 2.

Data analysis was performed in GraphPad Prism version 8 software. Two-way ANOVA followed by Tukey’s multiple comparison test was used for the human fibroblast analysis (three genotypes, four time points), the HEK293 cell analysis (three genotypes, four different treatments) as well as for autophagic-lysosomal impairment of HEK293 cells (two genotypes, two different treatments). A Kruskal-Wallis rank sum test followed by Dunn’s multiple comparison test was used for statistical analysis of human frontal cortex tissue in AD versus control cases (soluble vs. insoluble fraction in each AD and control) and for mouse brain tissue analysis (three genotypes, combined sexes).

## Supplementary Material

Supplemental Material
