# Supplementary material for: Sensitive ELISA-based detection method for the mitophagy marker p-S65-Ub in human cells, autopsy brain, and blood samples
Source: Autophagy. 2020 Oct 28;17(9):2613–28. doi: 10.1080/15548627.2020.1834712 (PMC8496550; doi:10.1080/15548627.2020.1834712)
Supplement: Supplemental Material [file KAUP_A_1834712_SM7522.zip › Supplementary information/Supplementary_Information_Watzlawik_R3.docx]

**Sensitive ELISA-based detection method for the mitophagy marker p-S65-Ub in human cells, autopsy brain, and blood samples**

Jens O. Watzlawik ^a^, Xu Hou ^a^, Dominika Truban ^a^, Chloe Ramnarine ^a^, Sandeep K. Barodia ^b^, Tania F. Gendron ^a, c^, Michael G. Heckman ^d^, Michael DeTure ^a^, Joanna Siuda ^e^, Zbigniew K. Wszolek ^f^, Clemens R. Scherzer ^g,^, Owen A. Ross ^a, c^, Guojun Bu ^a, c^, Dennis W. Dickson ^a, c^, Matthew S. Goldberg ^b, h^, Fabienne C. Fiesel ^a, c^, Wolfdieter Springer ^a, c *^

*^a^ Department of Neuroscience, Mayo Clinic, Jacksonville, FL 32224, USA*

*^b^ Center for Neurodegeneration and Experimental Therapeutics, The University of Alabama at Birmingham, AL 35294, USA*

*^c^ Neuroscience PhD Program, Mayo Clinic Graduate School of Biomedical Sciences, Jacksonville, FL 32224, USA*

*^d^ Division of Biomedical Statistics and Informatics, Mayo Clinic, Jacksonville, FL 32224, USA*

*^e^ Department of Neurology, Medical University of Silesia, Katowice, Poland*

*^f^ Department of Neurology, Mayo Clinic, Jacksonville, FL 32224, USA*

*^g^ Center for Advanced Parkinson Research, Harvard Medical School, Brigham and Women’s Hospital, Boston, MA 02139, USA*

*^h^ Department of Neurology, Department of Neurobiology, The University of Alabama at Birmingham, AL 35294, USA*

** Corresponding author*

**Correspondence should be addressed to:**

Wolfdieter Springer, PhD, Department of Neuroscience, Mayo Clinic, 4500 San Pablo Road, Jacksonville, FL 32224, USA. Tel.: +1-904-953-6129; Fax: +1-904-953-7117; Email: [Springer.Wolfdieter@mayo.edu](mailto:Springer.Wolfdieter@mayo.edu)

**Running title:** Development of a highly sensitive p-S65-Ub sandwich ELISA

**SUPPLEMENTARY MATERIAL**

**Supplementary figure legends**

**Figure S1.** Specificity of p-S65-Ub antibodies toward phosphorylated Ub. Detection of recombinant phosphorylated Ub monomers and poly-Ub chains with different linkages was compared on western blots. Cross reactivity of all p-S65-Ub Abs (A-D) with recombinant p-S65-PRKN was determined and only Ab B detected phosphorylated PRKN protein similar to a p-S65-PRKN control antibody.

**Figure S2.** p-S65-Ub sandwich ELISAs using different total Ub detecting Ab concentrations. Rabbit p-S65-Ub Abs (A-D) (1 μg/ml each) were used as capturing agents and each combined with three different concentrations of detecting Ab Ub 2 (0.2, 1.0, or 5.0 μg/ml). Recombinant p-S65/total Ub monomers and tetramers with different chain linkage were tested in identical mass concentration (33.3 ng/ml). Each graph represents a different p-S65-Ub Ab (A-D) combined with different concentrations of detecting Ab Ub 2. Data points are shown as MSD ECL (mean + Std. Dev.) from three technical replicates.

**Figure S3.** Autophagic-lysosomal impairment in WT and *PINK1* KO HEK293 cells. p-S65-Ub levels were assessed by sandwich ELISA using p-S65-Ub Ab D (1 μg/ml) combined with total Ub Ab 2 (5 μg/ml) using 10 μg total protein for HEK293 cells. WT HEK293 cells and *PINK1* KO HEK293 cells were treated with 400 nM bafilomycin A_1_ or vehicle control (DMSO) for 24 h. Data points are shown as MSD ECL (mean + Std. Dev.) from three replicates. Asterisks (*) indicate comparison to bafilomycin A_1_-treated WT HEK293 cells, plus signs (+) refer to comparisons between DMSO-treated WT HEK293 cells and *PINK1* KO HEK293 cells. Two-way ANOVA combined with Tukey’s test for multiple comparisons (***/+++ p < 0.0001, n.s non significant).
