# Supplementary figures and images for: Sensitive ELISA-based detection method for the mitophagy marker p-S65-Ub in human cells, autopsy brain, and blood samples
Source: Autophagy. 2020 Oct 28;17(9):2613–28. doi: 10.1080/15548627.2020.1834712 (PMC8496550; doi:10.1080/15548627.2020.1834712)

Supplementary Figure 1

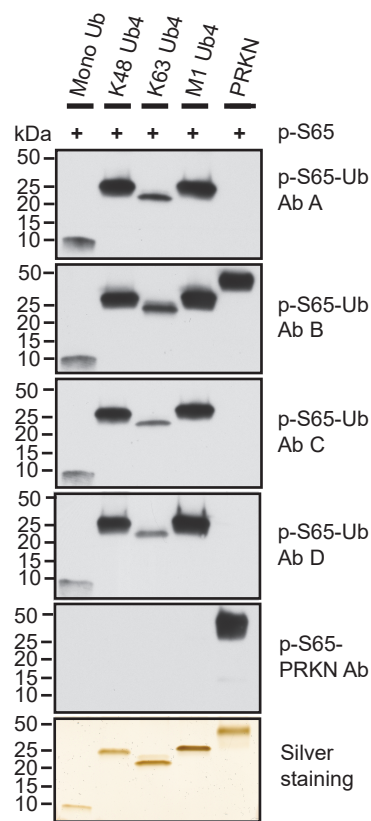

Supplement: Supplemental Material [file KAUP_A_1834712_SM7522.zip › Supplementary information/SFigure_1.pdf]

Supplementary Figure 2

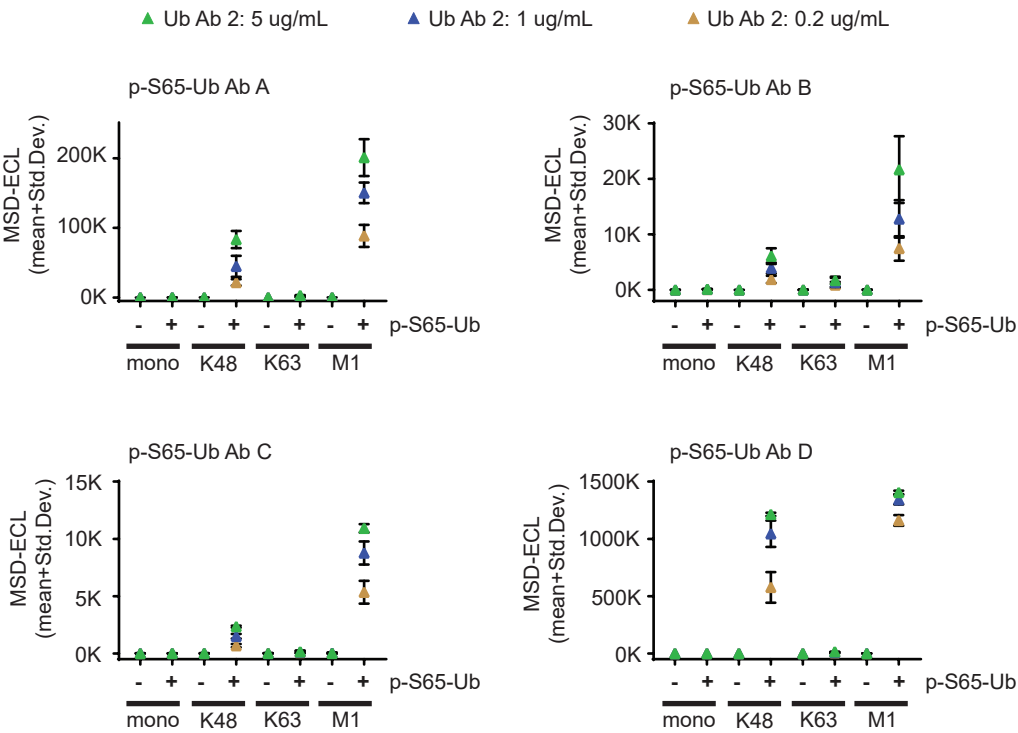

Supplement: Supplemental Material [file KAUP_A_1834712_SM7522.zip › Supplementary information/SFigure_2.pdf]

Supplementary Figure 3

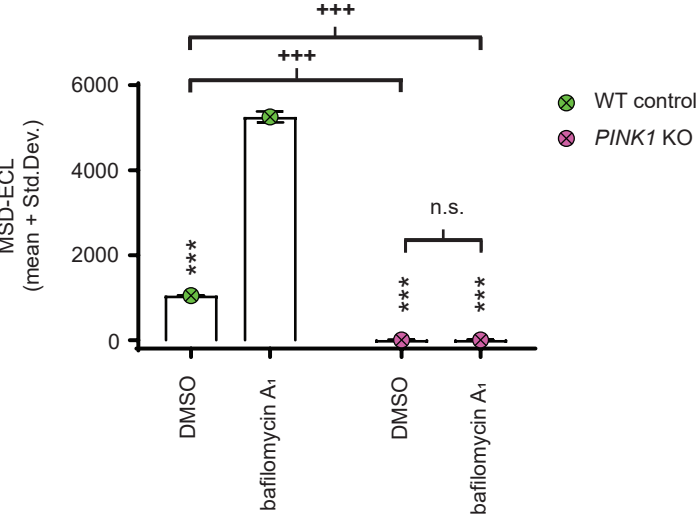

Supplement: Supplemental Material [file KAUP_A_1834712_SM7522.zip › Supplementary information/SFigure_3.pdf]
